# Review: Mechanisms of Glyphosate and Glyphosate-Based Herbicides Action in Female and Male Fertility in Humans and Animal Models

**DOI:** 10.3390/cells10113079

**Published:** 2021-11-08

**Authors:** Loïse Serra, Anthony Estienne, Claudine Vasseur, Pascal Froment, Joëlle Dupont

**Affiliations:** 1 CNRS, IFCE, INRAE, Université de Tours, PRC, F-37380 Nouzilly, France; loise.serra@inrae.fr (L.S.); anthony.estienne@inrae.fr (A.E.); pascal.froment@inrae.fr (P.F.); 2 Assisted Medical Procreation, Pôle Santé Léonard de Vinci, F-37380 Chambray-lès-Tours, France; claudine.vasseur@inrae.fr

**Keywords:** glyphosate, roundup, fertility, endocrine disruptors, steroidogenesis, hypothalamic-pituitary-gonadal axis

## Abstract

Glyphosate (G), also known as *N*-(phosphonomethyl)glycine is the declared active ingredient of glyphosate-based herbicides (GBHs) such as Roundup largely used in conventional agriculture. It is always used mixed with formulants. G acts in particular on the shikimate pathway, which exists in bacteria, for aromatic amino acids synthesis, but this pathway does not exist in vertebrates. In recent decades, researchers have shown by using various animal models that GBHs are endocrine disruptors that might alter reproductive functions. Our review describes the effects of exposure to G or GBHs on the hypothalamic–pituitary–gonadal (HPG) axis in males and females in terms of endocrine disruption, cell viability, and proliferation. Most of the main regulators of the reproductive axis (GPR54, GnRH, LH, FSH, estradiol, testosterone) are altered at all levels of the HPG axis (hypothalamus, pituitary, ovaries, testis, placenta, uterus) by exposure to GBHs which are considered more toxic than G alone due to the presence of formulants such as polyoxyethylene tallow amine (POEA).” In addition, we report intergenerational impacts of exposure to G or GBHs and, finally, we discuss different strategies to reduce the negative effects of GBHs on fertility.

## 1. Introduction

Glyphosate (G), also known as N-(phosphonomethyl)glycine, is a broad-spectrum herbicide used in agriculture. It was first synthetized by the chemist Henry Martin in 1950 from a derivative of the amino acid glycine and used in herbicide formulations only two decades later by John Franz (Monsanto) [1]. Glyphosate-based herbicides (GBHs) are commonly composed of 36–48% G, water, salt, and formulants such as polyoxyethylene tallow amine (POEA), an ethoxylated alkylamine, heavy metals or polycyclic aromatic hydrocarbons (PAHs), and quaternary ammonium [2,3]. However, the composition of formulants is often unknown because of the confidentiality of the mixture. The most known GBH is Roundup (R), produced by Monsanto. Currently, G can have non-agricultural applications such as residential and industrial uses, including the control of plants on roads and in cocaine and marijuana plantations [4]. In agriculture, plants tolerant to G have been developed; therefore, G can operate against 100 species of weeds and about 60 species of perennial weeds [1,4]. G acts on the shikimate pathway, which exists in bacteria, archaea, fungi, and algae, for aromatic amino acids synthesis. It inhibits 5-enolpyruvylshikimate-3-phosphate synthase (EPSPS), which does not exist in vertebrates. Thus, by interrupting aromatic amino acid metabolism, G drives plants to death [5]. After its use on plants, G is still found in soil and water, where its half-life is from 2 to 197 days and 91 days, respectively [1]. It is transformed by glyphosate oxidoreductase into glyoxylate and amino-methyl-phosphonic acid (AMPA) [1,4]. In a study of intoxication to G by ingestion, serum levels were quantified at 22.6 mg/L for G and 0.18 mg/L for AMPA after 8 h, and 4/4 mg/L and 0.03 mg/L respectively after 16 h. The detection of both G and AMPA was possible until the fourth day of hospitalization. The ratios of serum concentration for G and AMPA were placed at 126:1 and 147:1, 8 h and 16 h, respectively, after ingestion, and 148:1 for G and AMPA found in urine suggesting few amounts of G metabolized to AMPA [6].

Many studies on G have been performed and different thresholds have been determined. In humans, the no observed adverse effect level (NOAEL) was identified at 50 mg/kg bodyweight/day (mg/kg/bw/d) in 2015. According to the European Food Safety Authority (EFSA), the acceptable daily intake (ADI) is 0.5 mg/kg/bw/d and the acceptable operator exposure level (AOEL) is 0.1 mg/kg/bw/d. In rats, the threshold for long-term toxicity was determined at 350 mg/kg/bw/d [5] and the median lethal dose (LD50) was 5 g/kg/bw/d [7]. The Food and Agricultural Organization of the United Nation (FAO) has analyzed the maximum residue limits (MRL) of different types of food and found a range from 0.025 to 2 mg/kg of G in meats, beans, and milk, and around 30 mg/kg of G in rice, wheat, and oats [4]. Analysis of urine levels in humans has shown that normal people can have less than 4 µg/L of G in their urine, whereas people living in areas where GBHs are widespread can have up to 7.6 µg/L of G [8]. The latter value is higher than the dietary daily intake value (DDI) found in urine, which is from 0.1 to 3.3 µg/kg/bw/d [5].

The above-mentioned studies have been conducted in the context of increased use of endocrine-disrupting chemicals (EDCs) in agriculture and for common uses (cosmetics, detergents, etc.). An EDC is “a synthetic chemical that, when absorbed by the body, either mimics or blocks hormones and disrupts the body’s normal functions” [9]. La Merrill et al. [10] identified 10 key characteristics (KCs) of EDC, including “antagonizes hormone receptors,” “alters signal transduction in hormone-responsive cells,” “alters hormone distribution or circulating levels of hormones,” or “induces epigenetic modifications in hormone-producing or hormone-responsive cells.” G is a good candidate for an EDC. Indeed, when employed in the R formulation, at 0.05%, it disturbed estrogen receptors (ERs), namely ERα and Erβ, in breast cancer. Moreover, it has an impact on cell survival, cell cycle, metabolism, reactive oxygen species (ROS) levels, and cell death, among other processes [4]. At 50 mM, G and its metabolite AMPA inhibited cell growth and decreased cell viability in the cancer cell lines SKOV-3 and OVCAR-3 [4]. In their review, Ingaramo et al. [11] discussed evidence that GBHs can be considered EDCs. They mentioned the capacity of GBHs to inhibit cell proliferation, to promote cell death in human ovarian cell lines, and to perturb the production of progesterone (Pg) and estrogen in bovine ovarian cells. GBHs have adverse effects, such as toxic effects on human blood and peripheral blood mononuclear cells (PBMCs) (1–100 µg/mL), increased permeability of the blood–brain barrier (100 µM), increased ROS levels in epithelial cells, and decreased cell viability of several cell lines, including HepG2 (a liver hepatocellular carcinoma cell line) and JEG3 (a human choriocarcinoma cell line). The authors also mentioned that deleterious effects of G were improved by formulants used in GBH formulations because G alone, at a low concentration, had less impact than in formulations with the same level of G [4,12]. Moreover, formulants alone are more toxic than G or GBHs [12]. Indeed, studies on formulants such as POEA and formulation such as Roundup have shown their toxicity for the cells. In Hao et al.’s study [13], the authors analyzed the mechanisms by how POEA in the formulation of R (G: 41%; POEA: 14.5%) altered lung A549 cells with a non-exhaustive list: the collapsing of mitochondrial membrane, the increase of apoptosis, the oxidative damages with DNA single-strand breaks and double-strand breaks whereas none of these effects were observed with G alone [13]. In another formulation (Roundup Bioflow in Italy or Roundup Ultra in Belgium) used in European countries and containing a propoxylated quaternary ammonium surfactant, residues were found in urinary excretion after 90 days-exposure in female Sprague Dawley rats. The formulation was given by drinking water with concentrations of ADI (0.5 mg/kg bw/d of G; 0.17 mg/kg bw/d of the surfactant), European NOAEL (50 mg/kg bw/d of G; 17 mg/kg bw/d of the surfactant) and US NOAEL (175 mg/kg bw/d of G; 58 mg/kg bw/d of the surfactant). The urinary residues concentrations found were 0.0078 mg/L, 0.19 mg/L and 3.29 mg/L, respectively. These data showed that the formulants contained in the R formulation can be found in the urine and correspond to absorption of around 0.1% of the amount ingested [14].

All these studies have focused on the impact of G or GBHs in different organs, but according to our knowledge, no review has been written about the impacts of G and/or GBHs on the hypothalamic–pituitary–gonadal (HPG) axis and how the effects are related. In this review, we first review studies that have examined how exposure to G or GBHs affects the hypothalamus, pituitary, and gonads. Then, we discuss investigations on how G or GBHs alter embryonic, placental, and uterine development; exert transgenerational effects through epigenetic modifications, and lead to reproductive pathologies. Finally, we report some strategies to avoid the adverse effects of G.

## 2. Hypothalamus

The hypothalamus is the first actor in the HPG axis. It enables neurons located in the preoptic area (POA) to release in a pulsatile manner gonadotropin-releasing hormone (GnRH) into capillaries that reach the anterior pituitary gland [15]. Located within GnRH neurons, GPR54 is the receptor for the protein kisspeptin (Kiss1), which regulates GnRH neurons. Rats with Kiss1 knockout (KO) showed inhibition of luteinizing hormone (LH) and follicle-stimulating hormone (FSH) secretion as well as a positive feedback of estrogen on LH secretion during ovulation. Moreover, Kiss1 regulates testosterone (T) secretion from pubertal onset to adulthood in males to induce masculinization [16]. Thanks to the binding of GnRH to its receptor, GnRHR, in gonadotrophs located into the anterior pituitary gland, FSH and LH are released and act on their receptors, FSHR in granulosa cells and LHR in theca and granulosa cells, respectively, in females, and FSHR in Sertoli cells and LHR in Leydig cells in males. Altogether, they trigger and regulate gametogenesis and steroidogenesis.

Cericato et al. [17] performed a study on jundiá (*Rhamdia quelen*) to evaluate the impact of R on the hypothalamic–pituitary–renal (HHR) axis. Fishes were exposed to different doses of pesticides (16.6%, 33.3%, and 50% of the LC50); then, the authors induced or did not induce stress and analyzed the cortisol secretion response. After exposure to R, they observed that 50% of the LC50 reduced the secretion of cortisol compared with the induced-stress control group and increased the secretion of cortisol compared with the control group (no stress-induced, no pesticide exposure). They concluded that R disturbed the HHR axis at 50% of the LC50 [17]. Another study showed that GBHs disturb the hypothalamic–pituitary–thyroid (HPT) axis. Experiments were run on female Wistar rats exposed to different concentrations of Roundup Transorb (0, 5, or 50 mg/kg/day) from gestational day 18 (GD18) to postnatal day 5 (PND5). In male offspring, this exposure dysregulated the expression of myriad genes coding for thyroid hormone transporters (Mct8 and Oatp1c1), thyroid hormone receptors (*Thra1* and *Thrb1*), and thyroid hormone enzymes catalyzing T4 and T3 conversion (*Dio2* and *Dio3*). These perturbations were correlated with hypothyroidism features and affected metabolism in the hypothalamus, pituitary, heart, and liver [18]. Therefore, GBHs certainly have an impact on the hypothalamus. Martinez et al. [19] went further and analyzed the effects of G on neurotransmitters in rat brain regions. Male Wistar rats were exposed to different doses (35, 75, 150, and 800 mg/kg/bw) for 6 days, and then their brains were removed and examined. There were diverse effects on the hypothalamus, with impacts on the levels of serotonin and its metabolites, dopamine and its metabolites, homovanillic acid and norepinephrine and its metabolites. These data suggest that G and other components of the formulation in GBHs can enter the brain through the blood–brain barrier and alter the serotonergic, dopaminergic, and noradrenergic systems [18,19].

### 2.1. Kiss Expression

Smith et al. [20] worked on Hd-rR strain medaka embryos to analyze whether G (0.5 mg/L) or R (0.5 mg/L or 5 mg/L) could alter the HPG axis. They exposed embryos from fertilization until day 15 of embryonic development and then waited until the embryos grew and reached sexual maturity (without further exposure to G or R). In adult females, they measured the messenger RNA (mRNA) level of the neuroendocrine hormones *Kiss 1* and *Kiss 2*, as well as their receptors, *Gpr54-1* and *Gpr54-2*, respectively. There were no significant changes in the *Kiss 1* and *Kiss 2* mRNA levels, but there was a downregulation of *Gpr54-1* and *Gpr54-2* at 0.5 mg/L of R. Therefore, R exerts effects on neurons involved in reproduction and the HPG axis. To our knowledge, no other studies have examined the effects of GBH exposure on Kiss proteins and their receptors. More investigations should be conducted into how G or GBHs could affect the HPG axis through Kiss regulation [20].

### 2.2. GnRH Secretion

Only two studies have been performed about the effects of G and GBHs on GnRH secretion. Fu et al. [21], performed an in vivo study on 4 groups of weaned female piglets fed with 4 different concentrations of R (0, 10, 20, and 40 mg/kg) for 36 days. They found that G increased the level of GnRH in serum [21]. Another study found that G and R (0.5%) exposure during pregnancy in mice, reduced *GnRH* gene expression in the hypothalamus [22]. These studies seem to be in opposition concerning the impact of G on GnRH secretion. However, the opposite results could be due to species specificities, the doses that were used within the experiments, and the time and duration of the exposure.

**Figure 1:**
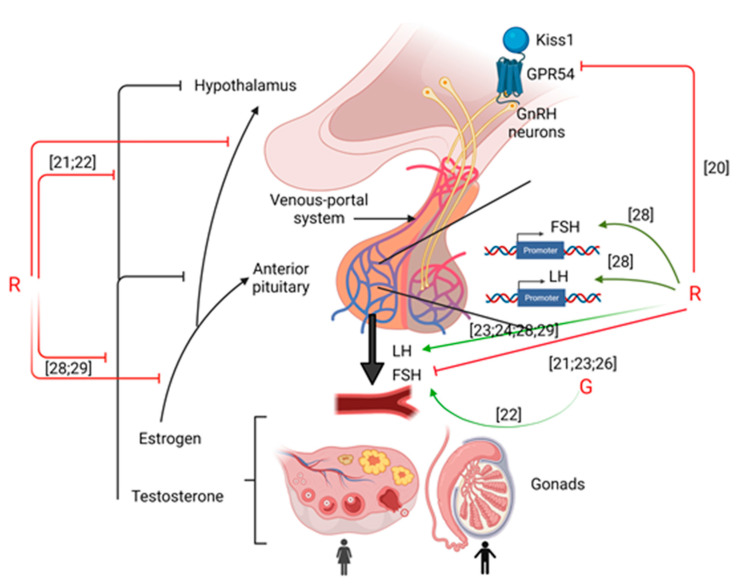
Summary of the effects of G or R on hypothalamus-pituitary-gonads axis. Green arrows mean activation and red arrows mean inhibition. Numbering in brackets indicates references.

Thus, despite the few amount of results, it appears that G or GBHs impact hypothalamic functions through the impairment of the Kisspeptin receptor and GnRH release (cf Figure 1).

## 3. Pituitary

Only a few studies have been performed to evaluate the effects of G or GBHs on the secretion of LH and FSH [18].

### 3.1. Long Term Exposure to GBHs

In 2018, Popoola et al. [23] studied the effect of R on male Wistar rats and showed that 400 and 2000 mg of G/kg/bw/d impacted the adenohypophysis; those doses correspond to 8 and 40 times the maximum based on the NOAEL, respectively. First, there was a decrease in plasma FSH secretion and an increase in plasma LH and prolactin secretion. Second, T was diminished whereas estradiol (E2), Pg, and the T/E2 ratio were increased. These effects were significantly higher with the 2000 mg/kg/bw/d dose [23]. The authors also examined the adenohypophysis histology. There was cellular hypoplasia with mitotic bodies, an increase in the amount of connective tissue, and a decrease in the number of cells. Astiz et al. [24] analyzed the effects of a combination of three pesticides known to influence oxidative balance and reproductive parameters: zineb (15 mg/kg/bw), G (10 mg/kg/bw), and dimethoate (15 mg/kg/bw). Then, they intraperitoneally administrated this pesticide mixture (PM) to male rats three times a week for 5 weeks. This PM increased FSH by 54% and LH by 90% in plasma compared with the control group. Interestingly, when PM was co-administrated with alpha-lipoic acid (LA), the basal levels of FSH and LH were recovered. A decrease in T was also observed, and similarly to the gonadotropin levels, the T level was restored by LA administration [24]. Abarikwu et al. [25] used another formulation containing G—the Bremont Wipeout herbicide—and exposed 20 6-week-old male rats to 5 mg/kg of G for 52 days. G had no significant effect on plasma LH and FSH levels. However, the plasma T level was decreased by about 50% compared with the control group [25].

The authors of these three above-mentioned studies analyzed the direct impact of GBHs on male reproduction in adult Wistar rats after 35–60 days of GBH exposure. Popoola et al. [23] demonstrated the effects of R and Astiz et al. [24] showed the effects of G alone or with chemicals added. The results obtained from these studies remain unclear: There was an increase in plasma FSH in one study (35 days) whereas the others (with a longer exposure of 52 and 60 days) showed non-significant results for the younger group (6 weeks old) and a decrease in FSH for the adult group. For the younger group, only G was used whereas for the adult group, R was used, supporting the results that formulants added to the R formulation could increase the effects of G on fertility. For LH secretion, two studies have shown an increase in the LH level in plasma and pituitary. In another model (adult male albino rats), Owagboriave et al. [26] analyzed the effects of three different doses of R (3.6, 50.4, and 248.4 mg/kg bw) with a longer exposure compared with the above-mentioned studies (12 weeks). They observed decreased FSH and LH in the blood for all tested doses [26]. A long-term in vivo chronic study on Sprague Dawley rats aged 5 weeks, showed that R exposure for 2 years by food (R tolerant NK603 genetically modified maize with R application) or by drinking water (from 50 ng/L of G in R formulation to 2.25 g/L) led to abnormalities of the pituitary in females with apparitions of adenomas, hyperplasia and hypertrophies certainly related to the perturbation of androgen/ balance [27].

Thus, a longer exposure duration with R could have a more prominent impact than G alone.

### 3.2. Gestational Exposure to GBHs

Other authors have examined the consequences of direct GBH exposure during gestation on male offspring. Romano et al. [28] showed that maternal exposure during gestation impacted the next generation of male Wistar rats. The level of LH mRNA and protein were increased in the pituitary after exposure of the mother to Roundup Transorb (50 mg/kg/bw), and the serum LH level in the offspring was also increased. *Fsh* mRNA was increased in the pituitary in males. Moreover, the serum levels of T and E2 were also increased, suggesting increased conversion of T to E2. Normally, when T increases, it negatively regulates LH production. In this study, because LH was also increased, there appears to be an impact on the feedback loop where inhibition of LH by T is not effective [28].

Teleken et al. [29] exposed C57Bl/6 mice from GD4 to the end of weaning (PND30). R (G 0.5%, equivalent to 1.85 g/L) impacted the HPG axis. Indeed, there was a delay in testis descent in males and the intratesticular T concentration was increased by 195% compared with the control group, although the plasma T concentration remained unchanged. Moreover, secretion of plasma LH was boosted as in the study by Romano et al. [28], and there was a marked increase in βLH in the pituitary, whereas the plasma FSH level did not change compared with the control group. These results support the hypothesis that the feedback loop of T is not efficient at the pituitary level [28] and that LH is increased by G when exposure happens during the early stages of rat development. Ren et al. [22] found that R and G increased Lh gene expression and G impacted Fsh gene expression within the pituitary of pregnant mice. Finally, Fu et al. [21] examined weaned female piglets. They found that 36 days of exposure to R impacted the HPG axis by inducing dysregulation of sex hormones. FSH levels were quantified in the serum and for all tested conditions (0, 10, 20, and 40 mg/kg), FSH was decreased compared with the control group [21].

As shown in Figure 1 and Table 1, GBHs exert more pronounced effects on the pituitary and pituitary hormone secretion than G alone. These effects include modulation of LH and FSH secretion. In addition, the feedback loop by which T secretion reduces LH secretion in a normal state seems to be inefficient in the pituitary gland after GBHs exposure. Moreover, GBHs apparently go from mother to offspring during gestation, and the offspring do not seem to be protected from the negative effects of GBHs on their own future fertility. Finally, researchers have shown that GBHs exert effects on the pituitary of multiple mammalian species, leading to the conclusion that mammals can be affected by exposure to GBHs.

## 4. Gonads

### 4.1. Ovary

The ovarian cycle begins at the onset of puberty. The signals that lead to the beginning of puberty remain unclear and could be enhanced by hormones secreted by adipose tissues such as leptin. Of note, insulin, fatty acids, and glucose also have an impact on the ovarian cycle. Adipose tissue is the most widespread endocrine organ within the body, a factor that makes it a principal target for EDC, such as G and GHBs [30]. Exposure to chemicals could affect the endocrine function of adipose tissue, leading to the onset of puberty issues.

#### 4.1.1. Steroidogenesis

Steroidogenesis is the main process that enables the production of steroid hormones, which can acutely regulate the HPG axis.

Studies on the effects of exposure to G or GBHs on mammalian reproduction have shown impacts on steroidogenesis. The exposure of mice to R (0.5% G equivalent) during pregnancy altered the production of serum Pg and decreased ovarian *Lhr*, *Hsd3b1,* and *Cyp19a1* mRNA expression. Similar results were found with G at the same dose; in addition, estrogen was increased and *Fshr* mRNA expression was decreased [22]. For Sprague Dawley rats, exposure during pregnancy and weaning did not alter plasma estrogen production [31]. Moreover, mice exposed to 2 mg/kg/d of G five times a week for 20 weeks did not show any differences in steroidogenesis compared with control mice [32]. On the contrary, long-term exposure of Wistar rat to a GBH (Kalach 360 SL, 126 mg/kg) for 60 days decreased ovarian estrogen levels [33].

**Figure 2:**
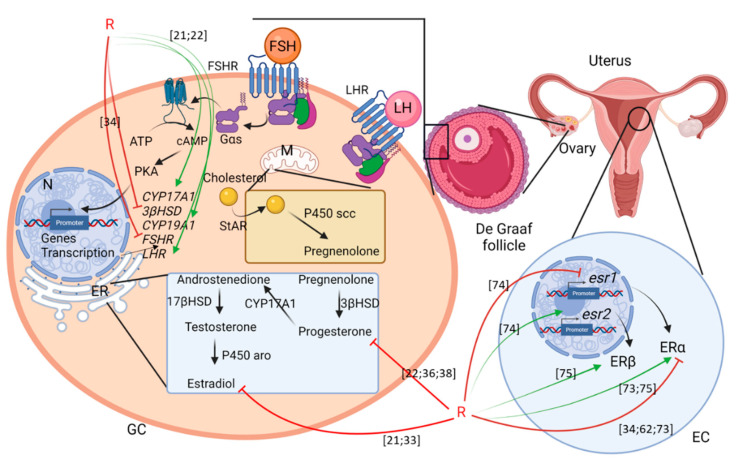
Scheme summering the effects of G or R on female fertility. GC: granulosa cells; EC: endometrial cells; ER: endoplasmic reticulum; M: mitochondria; N: nucleus. Green arrows mean activation and red arrows mean inhibition. Numbering in brackets indicates references.

In large mammals such as the pig, exposure of weaned piglets to R (10 mg/kg) for 36 days decreased the serum estrogen level and increased the serum T level [21]. Another study on Friesian sheep showed that ewes exposed to R (2 mg/kg/day) for 15 days during their first 15 days of life had decreased *Fshr* and *Gdf9* mRNA expression, which is essential for *Fshr* expression [34]. All the doses used were below the NOAEL (50 mg/kg/bw) (Figure 2, Table 2). By using in vitro experiments, the exposure of bovine ovarian cells to R (10 mg/L) for 2 days decreased estrogen and Pg production; however, 1 mg/L of R increased estrogen and Pg levels in the presence of insulin-like growth factor 1 (IGF1), FSH, and T. Without IGF1, estrogen levels were decreased [35]. Interestingly, 5 mg/L of G decreased the estrogen level but not the Pg level, and 10 mg/L of G did not change the expression of steroid hormones [36]. Bovine ovarian cells collected and cultured in vitro respectively at day 8 and 12 of the estrus cycle were exposed to 10 µg/L of R, which had no impact on steroidogenesis [37]. In swine cells exposed to 200 µg/L of G for 2 days, granulosa cells showed decreased estrogen production and increased Pg production [30]. In other studies, 2 days of R exposure at 360 mg/L in porcine cumulus-oocyte complexes (COCs) led to a decrease in Pg [11,38].

Overall, there is a real impact of G or GBHs on ovarian steroidogenesis. This impact seems to be different depending on the doses used and the time of exposure as well as the species studied. Finally, G and GBHs seem to affect steroidogenesis, mostly within granulosa cells, by altering estrogen and Pg synthesis (Figure 2 and Table 3).

#### 4.1.2. Ovarian Alterations

##### In Vivo Studies

In vivo studies have been performed on different animal models from *Danio rerio* (zebrafish, a teleost) to large mammals such as ewes to analyze the toxic effects of G or GBHs on ovaries. First, regarding their impact on the weight of ovaries, G and GBHs do not have the same effect. Indeed, G exposure at 2 mg/kg five times a week for 20 weeks in 6-week-old C57BL6 mice increased the weight of ovaries [32], whereas there was a decrease in the weight of ovaries when mice were exposed during pregnancy or during long-term exposure to GBHs. This latter result was also associated with an increase in the number of atretic follicles and an alteration in the theca interna structure [22,33].

Regarding the follicle population, in D. rerio, exposure to Roundup WG at 65 µg/L for 15 days led to an increased number of initial follicles and a decreased number of intermediate and tertiary follicles compared with the control group [41]. Similar results were found with Roundup Full II^®^ Argos in Friesian ewes exposed during the first day of life, with an increase in granulosa cell apoptosis [34]. However, when G was used in 6-week-old C57BL6 mice for a long period, there were more primordial, primary, secondary, and antral follicles [32]. By contrast, a high dose of G during pregnancy decreased the number of mature follicles, a phenomenon associated with an increase in atretic follicles [22]. Regarding ovarian cell proliferation and viability, Alarcon et al. [34] showed decreased ovarian cell proliferation associated with more multiple-oocyte follicles in Friesian ewes exposed to a GBH.

Other alterations in ovaries exposed to GBHs have been observed, such as atrophy, fat deposition, vasodilatation, and cell calcification [21]; concentric membrane formation [39,41]; vacuolization [33,41]; first estrus delay in rats [31]; fragmented nuclei; condensed chromatin [42]; and an increase in fibroblasts and MEC [22]. These results show that GBHs, which contain G and formulants, have more toxic effects on ovarian cells than G alone, no matter the type of GBH, the development stage and duration of exposure, and the species. These findings are in accordance with the results obtained in the study performed by Defarge et al. [12] on the different effects of G, GBHs, and formulants.

##### In Vitro Studies

In vitro studies have demonstrated a negative impact of G and GBHs on granulosa cell proliferation, with GBHs showing the greater impact [30,35,36,42]. No significant impact on the viability of bovine cells exposed to G was found [36], whereas the viability of granulosa cells decreased in a swine model [30]. Moreover, bovine ovarian cells exposed to R had reduced viability [35]. Finally, the results regarding alterations in ovaries can be related to a perturbation in oxidative stress equilibrium, with an increase in nitric oxide (NO) [30], an increase in malondialdehyde (MDA) [22], a decrease in catalase (CAT) and superoxide dismutase (SOD) activities [22,33,42], and an increase in ovarian peroxidation [33,42] leading to an increase in ROS levels [11,38]. To our knowledge, no researchers have focused on the impact of G or GBHs on follicular fluid composition.

In conclusion, it is apparent that G and GBHs exert multiple adverse effects on ovarian function at different levels. They induce disturbances, at the molecular level, with dysregulation of steroidogenesis, leading to hormonal imbalance. At the cellular level, they can decrease cell proliferation and viability but also induce apoptosis. Finally, at the organ level, these chemicals can induce ovarian malformations, vascular modifications, or adipose tissue deposition. These effects are of course dependent on the doses used, the considered species, and the development stage and duration of exposure.

### 4.2. Testis

Within the testis, seminiferous tubules are the site of sperm production. Inside seminiferous tubules, Sertoli cells are present to support germ cells and extend the entire length of the germinal epithelium. They have a nutritional function for germ cells, as well as a physical support function and a role in germ cell cohesion. Between seminiferous tubules, interstitial tissue is composed of blood and lymphatic vessels, nerves, and Leydig cells, which are the main producer of steroid hormones within the testis [43]. Leydig cell functions are regulated mainly by LH secretion [15]. During mammalian in utero development, a major step for testis maturation is testicular descent. Leydig cells orchestrate this phenomenon with the help of the hormone insulin-like 3 (Insl3) and T production [29]. In males, T production and secretion are important for sexual differentiation, the development of secondary sexual characteristics, and the development of germ cells. Sertoli cells are the main physical support of germ cells. Moreover, Sertoli cells ensure the formation and maintain the integrity of the blood–testis barrier (BTB) [44] and the negative feedback regulation on pituitary and hypothalamus that downregulates LH and GnRH production [28]. Therefore, EDCs such as G can impair Leydig cell functions [28].

#### 4.2.1. In Vivo Studies

In Wistar rats, data have shown a decrease in serum T concentrations in males after puberty when mothers had been exposed to R during gestation (50–450 mg/kg/bw/d) [45]. Romano et al. [46] observed similar results when male Wistar rats were exposed during the prepubertal period to R (5–250 mg/kg/bw/d) for 30 days. These data are in good agreement with findings from other studies focusing on R [26,47] and G [23,24,25,48] exposure. Interestingly, in two studies with two different models, exposure from gestational to weaning in Wistar rats—Roundup Transorb (50 mg/kg/bw/d) [28]—and in C57Bl/6 mice—Roundup Original DI (1.85 g/L) [29]—triggered an increase in the serum and testis T concentrations, respectively, in adults. Studies performed on adult Sprague Dawley rats exposed to R or G at doses from 20 times lower to 10 times higher than the NOAEL [7] did not highlight specific changes in T secretion [44,49,50,51]. In other species such as Swiss mice, quails, drakes, pigs, and rabbits [52,53,54,55], a decrease in the T level was observed, regardless of the exposure period (gestation, adult, etc.), with doses from 56 µg/kg in rabbits [56] to 202 mg/kg/bw/d in pigs [52] of different GBHs (G alone, Roundup^®^ 3 plus, Roundup Flex, WILLOSATE, etc.).

Through the analysis of these studies, it appears that the developmental stage and the duration of exposure impact T secretion, with an increase when the exposure is performed during gestational and early postnatal stages and a decrease when exposure is performed at peripubertal stages. These effects are different depending on the GBHs. This last observation is in concordance with Defarge et al. [12], who showed that the composition of GBHs was important and influenced the endocrine disruption power of G. Disturbances of T secretion to impair E2 secretion (increased T conversion into E2), and so have a negative impact on the feedback loop on LH production [28,29] and in testis descent during in utero development [29].

What are the molecular mechanisms involved in the effects of G or GBHs on the Leydig cell steroidogenesis? StAR is important for the beginning of steroidogenesis with the entrance of cholesterol into the inner membrane of mitochondria. Exposure of adult BALB/c wild-type mice to G (5 g/L) for 4 weeks triggered a reduction in *Star* mRNA and protein expression [57], whereas exposure for 2 weeks (2.5–25 mg/kg/bw/d) in Wistar rats did not impact StAR production [51]. Zhao et al. [57] hypothesized that G impacts the circadian clock, denoted by increased nuclear receptor subfamily 1 group D member 1 (*Nr1d1*) expression, a change that would inhibit *Star* transcription and consequently decrease T production. Nr1d1, also known as Rev-erb-alpha, belongs to the family of “orphan receptors” and functions as a member of the clock gene family. Therefore, StAR seems to be altered by a high concentration of G. This decrease in StAR at high doses coincides with the results of a study performed in a pig model with a high dose of WILLOSATE, a GBH (103–202 mg/kg/bw/d), showing an increase in the cholesterol concentration [52]. P450scc, one of the first actors of steroidogenesis in mitochondria encoded by the *Cyp11a1* gene, was not impacted by G exposure at mRNA and protein levels (0.5–25 mg/kg/bw/d) during gestation in Swiss mice [54] and adulthood in Wistar rats [51]. The enzyme 3βHSD ensures the conversion of pregnenolone into Pg but also the conversion of DHEA into androstenedione, the substrate necessary for the production of T. In in vivo studies, it has been reported that *Hsd3b1* transcript and protein and *Cyp17a1* mRNA were decreased by G (5 g/L of G) [24,57]. However, an increase in Pg (400 mg/kg/bw/d of G)—the substrate of *Cyp17a1*, which converts it into 170H-Pg and then into DHEA—was observed in Wistar rats [23]. T and aromatase have a very close relationship. During gestation in rats, aromatase, encoded by the *Cyp19a1* gene, enables the conversion of circulating T into E2 in the central nervous system; the change then regulates the gender reproductive behavior in adults [11,28]. Increased *Cyp19a1* expression was observed after R exposure at 2.25 g/L for 8 days in 60-day-old Sprague Dawley rats [49]. Moreover, the product of aromatase, E2, was increased in adults when rats were exposed to G during gestation [23], with a decrease in T, or during adulthood, with an increase in T following R exposure [28] from 50 to 2 g/kg/bw/day, respectively. However, a lower concentration of R (5 mg/kg/bw/d) reduced E2 in adult drakes, a phenomenon associated with a decreased serum T level [53].

**Figure 3:**
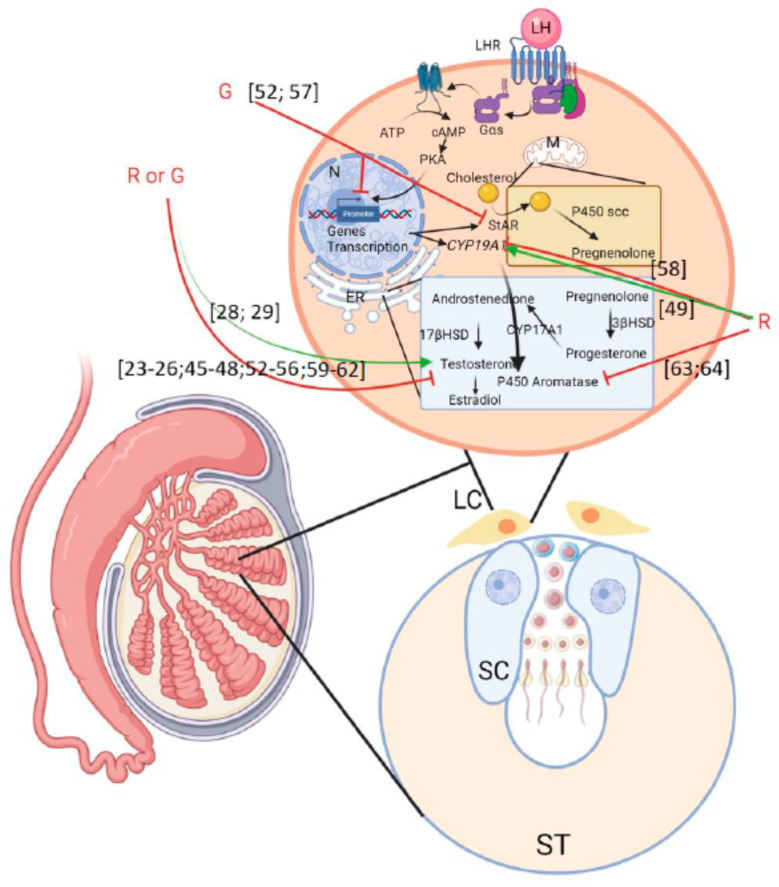
Scheme summering the effects of G or R on male fertility described in the literature. LC: Leydig cells; ST: seminiferous tube; SC: Sertoli cells. Green arrows mean activation and red arrows mean inhibition. Numbering in brackets indicates references.

Finally, focusing on Leydig cell damage, the number of these cells was decreased in association with hypertrophy and cytoplasmic granulations after G exposure (125 mg/kg/bw/d) for 30 days in 8-week-old Wistar rats [48]. Overall, steroidogenesis in Leydig cells is impacted by G and GBHs. In most studies, a decrease in T is observed regardless of the physiological stage and this impacts E2 production (Figure 3 and Table 4, Table 5 and Table 6). At the molecular level, the expression of StAR, 3βHSD, Cyp17a1, and Cyp19a1 are frequently altered by G and GBHs.

#### 4.2.2. In Vitro Studies

In vitro studies have also confirmed some alterations in T production in response to exposure to G or GBHs. Indeed, T secretion was decreased in primary Sprague Dawley rat testis cells after 24 h of stimulation with Roundup^®^ Bioforce or G at 360 µg/L, a dose 10,000 times lower than the ones used in conventional agriculture [58,63]. In this study, the Cyp19a1 mRNA level was decreased after exposure of cells to 3.6 mg/L R for 24 h, with no impact on Er (Estrogen Receptor) mRNA and Hsd3b1 enzyme expression. However, higher doses of R—from 180 mg/L to 3.6 g/L—increased apoptosis, membrane degradation, and necrosis of Leydig cells, whereas G alone only impacted apoptosis and decreased cell viability [57,58]. In another study using Leydig cells from Sprague Dawley rats, 135 mg/kg/bw/d of Roundup GT plus exposure for 8 days decreased E2 but not T levels [64]. Studies on the TM3 mouse Leydig cell line exposed to G alone from 5 to 16.9 mg/L showed a great impact on the expression of steroidogenic genes, with a decrease in *Cyp17a1* mRNA, *Cyp11a1* mRNA, and *Star* mRNA and protein levels, but no effect on *Hsd3b1* mRNA and protein levels [57,59]. Walsh et al. [60] confirmed the decreased StAR protein expression but not the decreased *Star* mRNA level. Thus, the authors concluded that R disturbed steroidogenesis by decreasing the post-transcriptional expression of StAR. Finally, Zhao et al. [57] found that inhibition of T production in response to G exposure could be explained by a reduction in *Star* transcription through an increase in *Nr1d1* mRNA and protein levels. In vitro studies on tissue explants from large mammalian models are rare, but in vitro explants of equine testis were cultured with high doses of Roundup Bioforce^®^ (2.16–7.2 g/L) and G (6.48 g/L), and the results showed a decrease in aromatase [61,62].

Overall, the data from in vitro studies are in accordance with the results obtained from in vivo studies. There is a great impact of G or different R formulations on steroidogenesis, with low doses (10,000 times lower than what is used for agricultural purposes) to high doses (agricultural use) affecting cell viability and membrane integrity and perturbing steroidogenesis. These changes are probably mediated through altered *Star* expression (Figure 3 and Table 5 and Table 6).

#### 4.2.3. Sertoli Cells

##### In Vivo Studies

As previously described, Sertoli cells are important for physical and mechanical support and nutrition of germ cells and they are also the main constituent of the BTB [44]. However, when Wistar albino rats were exposed to 375 mg/kg/bw/d of G (Knockdown 48 SL) for 8 weeks, there was degeneration of Sertoli cells [65]. Interestingly, exposure to R or G did not impact cell junctions between Sertoli and germ cells in Sprague Dawley rats exposed to 2–50 mg/kg/bw/d.

##### In Vitro Studies

Sertoli cell alterations seem to be more important due to exposure to R formulations rather than G alone. Indeed, treatment of Sertoli cells obtained from Sprague Dawley rats with 10 mg/L of R for 30 min increased the phosphorylation of mitogen-activated protein kinases (MAPKs), namely P38-MAPK and extracellular signal-regulating kinases 1/2 (ERK1/2). The same experiment performed for 48 h inhibited the T effects on BTB integrity, and 100 mg/L for 48 h redistributed claudin 11—a protein crucial for tight junctions—and so altered the BTB, whereas G alone had no impact on p38 MAPK and ERK1/2 phosphorylation but inhibited the T effects on the BTB [44] Moreover, oxidative stress was increased in the TM4 mouse Sertoli cell line and primary Wistar rat Sertoli cells. With 3.6 mg/L of G or R, succinate dehydrogenase activity was increased [63]. Moreover, 36 mg/L of R increased SOD and CAT activities [66]. These results are in accordance with increased apoptosis and necrosis in response to the R treatment at 36 mg/L, also associated with cell dissociations and decreased cellular proliferation [63,66]. G has been shown to activate protein kinase C (PKC) and p38 and ERK1/2, which trigger the release of Ca^2+^ within the cytosol. The increased Ca^2+^ leads to the opening of L-type-voltage-dependent Ca^2+^ channels and enables the Ca^2+^ to enter the cells and target inositol triphosphate (IP3) in the endoplasmic reticulum. IP3 increases the level of intracellular Ca^2+^, which at high doses activates cell death [23]. The increased intracellular Ca^2+^ was observed in cells of the testis, including Sertoli cells, and was associated with increased protein kinase C (PKC), phosphoinositide 3-kinase (PI3K), ERK1/2, and p38 MAPK phosphorylation after exposure to 36 mg/L R, a dose that is 100 times lower than those found in conventional agriculture [66].

In conclusion, G or GBHs alter Sertoli cells by increasing the oxidative stress and the intracellular Ca^2+^ content through activation of PKC–PI3K and p38 MAPK–ERK1/2 signaling pathways. These processes lead to apoptosis, disruption of the membrane, and reduced BTB integrity, essential for germ cell protection.

#### 4.2.4. Germ Cells Alterations

Germ cells are very sensitive cells within the testis; they are supported and surrounded by Sertoli cells. During spermatogenesis, followed by spermiogenesis, they become spermatozoa. These processes are finely regulated by local factors, steroids, and could be affected by EDCs. Sperm parameters are important to evaluate the quality of spermatozoa and thus male fertility. The most analyzed parameters are the concentration, viability, and the number of abnormal spermatozoa (head or tail defects) as well as the motility of the spermatozoa [54].

##### In Vivo Studies

The number of spermatozoa decreased when rats were exposed to Roundup Transorb (5–250 mg/kg/bw/d) or Roundup Monsanto (50 mg/kg/bw/d) during gestation and the prepubertal period [45,46]. This phenomenon was also observed in adult rats exposed to G or GBHs [25,26,48,50,65]. No impact on sperm motility was observed in Sprague Dawley rats exposed to the NOAEL (50 mg/kg/bw/d) or higher doses of Roundup at different developmental stages [44,49], whereas decreased sperm motility was observed in adult Wistar rats, mice, and pigs with exposure to G or R (5–125 mg/kg/bw/d) [25,26,48,52,54,67]. Based on these studies in different species, G and GBHs do not have the same impact on sperm motility. However, considering sperm morphology, most of the studies have shown an increase in abnormal sperm in rats, mice, pigs, and rabbits from when animals are exposed to 3.6 mg/kg/bw/d [25,26,45,47,48,49,52,56,65,67]. This altered sperm morphology was accompanied by decreased cell viability from immature (spermatogonia) to mature (spermatozoa) stages when exposure occurred during gestation [44,45,49] or adulthood [25,52,54,56,67] in rats, mice, pigs, and rabbits. Finally, lipid accumulation in germ cells was observed in Wistar rats exposed to high doses of G [48], as well as a decreased germinal epithelium height in prepubertal rats exposed to R [28].

##### In Vitro Studies

**Figure 4:**
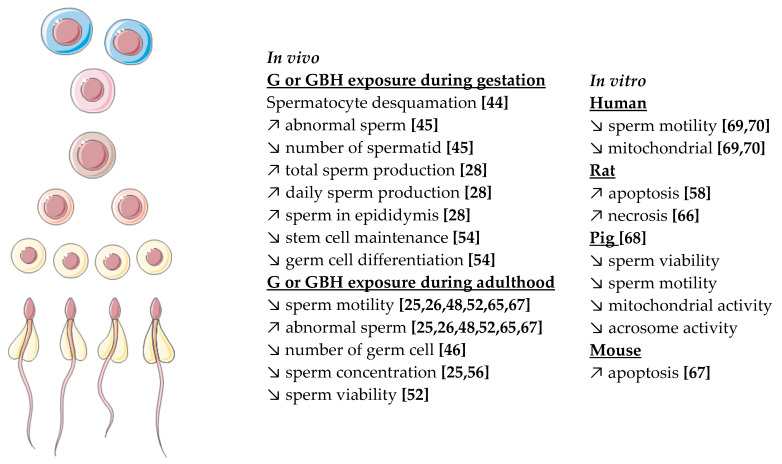
Summary of G or R in vivo and in vitro effects observed on spermatogenesis in different species. Numbering in brackets indicates references.

In accordance with in vivo studies, a decrease of sperm viability and motility was observed in the rat model after the exposition of sperm to R or to G alone (with doses three times higher than R) [68]. Human sperm motility was also affected by R at 360 µg/L, a dose that is 10,000 times lower than doses used in conventional agriculture [69,70]. This negative effect was accompanied by a decrease in mitochondrial membrane potential (MMP), mitochondrial activity, and mitochondrial respiration [67,68,71]. Mitochondria are a key organelle for spermatozoa, which enables motility, hyperactivation, capacitation, acrosome reaction, and finally fertilization. Crosstalk between mitochondria and steroid hormones exists and it is important for the maintenance of energy needed for sperm motility. The main process for energy production is the transformation of ATP by oxidative phosphorylation [71]. The decrease of sperm motility observed after G or GBH exposure is certainly due to the decrease of mitochondrial activity. Moreover, a decrease in acrosome integrity was observed, supporting the important role of mitochondria for acrosome reaction [68]. Finally, disruption of mitochondria was accompanied by an increase in sperm apoptosis and increased oxidative stress (cf Figure 4) [58,66,67].

#### 4.2.5. Other Alterations of Male Reproductive System

The testis weight was decreased after exposure to G (125 mg/kg/bw/d) for 10 days in adult Wistar rats [48]. Similar results were obtained in Swiss mice when fetuses were exposed to a low dose (1/100 of the NOAEL) of G or Roundup 3 plus [48,54] and a high dose of WILLOSATE [52]. Seminal vesicle weight was also impacted by exposure to G and Roundup [28,50,54]. G or GBHs have been reported to decrease the global epididymal weight in adult Wistar rats exposed during gestation to Roundup Transorb or during adulthood to G alone [25,28]. To our knowledge, no impact on the prostate gland has been recorded [47]. Tissue alterations have also been observed for the testis and epididymis in ducks [53] and mice [67] after R exposure, with the presence of vacuoles, lipid droplets, and lysosomal granulation [45,53,54]. The BTB integrity is important for the correct development of germ cells and their protection against pathogenic agents and cells from the immune system. The BTB is mainly regulated by T and Sertoli cells [44]. Disorganization of the BTB epithelium, low cell adhesion, and increased BTB permeability associated with an increase in occludin were observed in Sprague Dawley rats exposed to 2 mg/kg/bw/d of R [44,49]. Finally, on a larger scale, puberty was delayed in Wistar rats exposed to R [46], and testis descent was also delayed in another study [29]. These deficits can be related to the fact that the expression of GPR54, the receptor of Kiss1 regulating T during puberty, was impacted by R [16,20].

In conclusion, the effects of GBHs or G alone are important in the reproductive system of males. They affect the male reproductive tract in different species at doses lower than the NOAEL, at the tissue and molecular levels in signaling pathways and steroidogenesis regulation. They disturb Sertoli cell and Leydig cell functions, which are essential for spermatogenesis and spermiogenesis, the processes required for germ cell development.

## 5. Uterus

### 5.1. In Utero and Perinatal Development

The mammalian uterus begins its development in utero, and for some species such as sheep, it finishes after birth. In lambs, the proliferation of endometrial glands, important for hormone secretion, happens during postnatal uterine morphogenesis, until PND56 [34]. Uterine cell proliferation is regulated by estrogen, Pg, and their receptors (Erα, Erβ, and PR), as well as transcription factors such as homeobox a10 (Hoxa10), forkhead box a2 (Foxa2), and the Wnt morphogens. In rodents, the uterus is divided into different parts: the uterine luminal epithelium (LE), where embryos implant; the endometrial stroma (SS), where decidualization occurs from the antimesometrial (AM) zone to the mesometrial (M) zone; and the glandular epithelium (GE) [72]. Female Wistar rats were exposed at PND1, PND3, PND5, and PND7 to 2 mg/kg/bw/d of Roundup Full II^®^, and their uteruses were observed at PND8 and PND21. Erα was increased in the SS at PND8 but decreased in the LE at PND21 compared with the control group. PR was also disturbed, with increased expression within the SS and the LE at PND8 and the SS at PN21. Moreover, several actors important for uterine growth and differentiation during embryogenesis and postnatal development were studied: Hoxa10 and Wnt7a [73]. Hoxa10 was induced within the SS at PND8, and within the myometrium and the LE at PND21. Wnt7a was increased within the SS and the GE at PND21. At PND60, esr1, coding for *Erα*, and esr2, coding for *Erβ*, were downregulated and upregulated, respectively, associated with cell proliferation leading to a hyperplastic uterus. *Wnt7a* mRNA and protein levels were also upregulated [74]. In the uterus of adult female rats exposed to G (0.5–50 mg/kg/bw/d) for 3 days, perturbations in *Erα*, *Erβ*, and *Pr* mRNA and protein levels were recorded, without any difference in terms of cell proliferation [75]. G and R at 10 µg/L had no impact on viability and proliferation of endometrial and myometrial luteal cells in cows at day 8 and 12 of the estrus cycle [37]. In Friesian ewes exposed from PND1 to PND14 to 2 mg/kg/bw/d of Roundup Full II^®^, there was decreased endometrial cell proliferation (in the LE, the GE, the SS, and the myometrium) at PND45 accompanied by the inhibition of the production of an important protein for gland development: Foxa2 [34,40]. Moreover, no serum E2 variations were observed but Erα was downregulated in the LE, the GE, and the SS. Interestingly, PR expression was decreased in the LE, whereas it was increased in the GE and the SS.

Therefore, exposure to G or GBHs during perinatal development alters steroid hormones regulation, uterine growth, and differentiation in different mammalian models.

### 5.2. Puberty

During puberty in women, the uterus undergoes the menstrual cycle, which comprises four phases: menstruation, the proliferative phase, the window of implantation, and the secretory phase, when E2 and Pg play a crucial role in uterine development [76]. An in vitro study on the human endometrial Ishikawa cell line that conserves ER and PR expression was performed with G exposure from 33.8 to 338.1 µg/L for 24, 48, and 72 h. The results showed that G decreased cell adhesion, denoted by inhibited *CDH1* gene expression (encodes E-cadherin), and increased cell invasion and migration. This result is in accordance with the hypothesis that G is involved in breast and uterine cancers, characterized by loss of cell adhesion and gain of cell migration and invasion [77,78]. The consequences of GBHs exposure on the uterus were not analyzed at this stage.

### 5.3. Peri-Implantation Period

After several days of development within the uterine lumen, the embryo at the blastocyst stage needs to implant into the uterus to continue its development, and this action requires a receptive endometrium. At this time, the endometrium undergoes morphological, biochemical, and genetic changes, all of which are possible thanks to the secretion of steroid hormones and the expression of their receptors. During the proliferative phase, estrogen secretion induces Erα, whereas Pg decreases Erα during the window of implantation, which inhibits cell proliferation [76]. Lorenz et al. [76] exposed female Wistar rats to 2 mg/kg/bw/d G or MAGNUM SUPER II from GD9 to weaning (F0 generation) and analyzed the reproductive performance of the F1 generation until GD19. They observed a decrease in the number of implantation sites and an increase in the pre-implantation loss for both the G and GBH groups. Steroid hormones were impacted too, with an increase in serum E2 levels at GD5 for the G and R groups but no change for the serum Pg level. In the uterus, the Erα protein level was increased (in the G and R groups) including in the SS (G), but the transcript levels were not altered. PR protein was not disturbed in the uterus, but *Pr* mRNA was decreased by G exposure [76].

The same experiment was performed by Guerrero Schimpf et al. [73], and pregnancies in the next generation were analyzed. At GD9, there were more endometrial sites with mass, and post-implantation losses were recorded. Erα was decreased in the GE, and PR transcript and protein levels were also decreased in the AM and the M. Moreover, the sites where decidualization happens were reduced. Ingaramo et al. also studied the effects of neonatal exposure to a glyphosate-based herbicide (GBH) on the reproductive performance and the molecular mechanisms involved in the decidualization process in adult rats. They found that the number of fetuses per dam did not change [72]. Molecular regulation was then analyzed. In general, Hoxa10 is also necessary for the secretory phase during endometrial receptivity, when it reaches its highest level in response to estrogen and Pg to enable endometrial differentiation [76]. This protein is found in nuclei of the SS and myometrial cells [11]. In the Ingaramo et al.’s study, Hoxa10 was induced in the SS at GD9, with inhibition of Wnt morphogens (Wnt5a and Wnt7a) after GBHs exposure [72]. At PND8, Wnt5a and β-catenin were increased in the LE and the SS, whereas at PND21, Wnt5a was increased in the LE and the LS, and β-catenin was increased in the LE and decreased in the SS. The cell cycle regulation was also altered with the inhibition of cyclins. These results explained the hyperplasia observed in the LE and the increasing thickness of the stromal and myometrial compartment at PND8 [11].

All these studies have shown dysregulation of steroid hormones and their receptors in the uterus, inducing altered cell proliferation, migration, and invasion in response to exposure to G or GBHs. As a result, G and GBHs certainly have a potential role in the pre-implantation loss of embryos and could be responsible for early miscarriages.

## 6. Placenta

Few studies have been performed to evaluate the effect of G and GBHs on the placenta. Most studies have been realized using in vitro models such as the human choriocarcinoma-derived placental JEG3 cell line. Various concentrations of Roundup Bioforce (from 36 mg/L to 7.2 g/L) were tested on these cells at different times. Twenty-four hours of G exposure (from 144 mg/L) inhibited aromatase activity and reduced E2 synthesis; the effects observed with R treatment were 3-fold greater than G alone [61,62]. Moreover, G toxicity was related to the increasing exposure time and all of the R formulations tested were more toxic in in vitro studies than G alone and activated apoptotic pathways [79]. In HUVEC C2519A (human primary cells from the umbilical cord) in vitro cultures, R also impacted cell viability; when POEA, one of the known formulants of Roundup [5], was tested, it had the most toxic impact on cells at lower doses compared with R or G. Indeed, POEA was added in the R formulation to increase cell permeability. The combination of POEA and G led to toxic effects, namely altered membrane integrity and mitochondrial respiration. The effect of the G metabolite, AMPA, had less of an impact on HUVEC and JEG3 cells compared with G and GBHs. Kongtip et al. [80] reported concentrations of 189 µg/L of G found in the serum of mothers at childbirth, which was higher than concentrations found in the umbilical cord serum (94.9 µg/L). An ex vivo human perfusion system experiment was carried out to analyze whether the placenta is permeable to G (33.8 mg/L). Caffeine and benzoic acid were reported to transfer throughout the perfusion system more easily than G. Only 15% of G crossed the placental barrier to reach the fetal circulation, with a very low permeability coefficient compared with caffein and benzoic acid [81]. Finally, a recent study on placental perfusion ex vivo from human full-term placenta analyzed the difference between G alone (1 ppm) and R formulation containing formulants such as POEA (1 ppm G equivalent) [82]. They showed that the fetal transfer rate was decreased by R from 30 to 120 min of exposure and fell under 20% of transfer after 150 min. Histology observations highlighted the destruction of fetal vessels. All these effects were not observed with G alone. This was in accordance with Defarge et al.’s study [12] which tested 5 co-formulants found in R formulation and demonstrated that in the JEG3 cell line aromatase activity was reduced after 24 h of treatment with POEA, POEA/F, and quaternary ammonium, all disturbed aromatase at a lower dose than R formulation and G alone [12]. These two last studies highlighted the fact that co-formulants had higher toxic properties than R or G alone and were considered endocrine disruptors.

Overall, G and GBHs are toxic to the placenta and umbilical cord cells at doses 100 times lower than those found in conventional agriculture (7.2 g/L). Although G has a limited ability to cross the placental barrier, it could reach fetal circulation and with formulants altered vessels.

## 7. Embryo Development

As demonstrated before, GBHs can reach uterine lumen and fetal circulation through the placenta and, so, they could probably induce changes in early or late embryo development.

### 7.1. In Vitro Studies

A study on the human embryonic kidney 293 cell line exposed to Roundup Bioforce for 72 h showed that 50% of cells degenerated within the first hours of exposure, with doses from 1.08 g/L. This result demonstrated the cytotoxic effect of R in human embryonic cells [61]. The toxic effect was time-dependent and dose-dependent; R was more toxic than G but less toxic than POEA [62,79]. Moreover, authors have found that the decreased cell viability was related to the disruption of translation of the mitochondrial enzyme succinate dehydrogenase, a phenomenon that impacted mitochondrial respiration [61,79]. Reduced estrogen was also observed within 24 h of treatment with 36 mg/L of R. Finally when researchers compared the effects of R on other cell types, embryonic cells appeared to be more sensitive compared with placental cells [61]. Alterations in microtubules and chromosomes were recorded in mouse metaphase II oocytes and embryos after 180 min of exposure to G, a phenomenon that was linked to zinc-mediated ROS production. At 8.4 mg/L, G already had a negative impact on pericentrin, a microtubule-organizing center (MTOC) component, as well as on intracellular zinc concentration in metaphase II oocytes and embryos. In addition, the increase in ROS formation was correlated with increased concentrations of G. These effects were more pronounced as the exposure time increased. This study confirmed the dual effect of G depending on the dose and the duration of exposure on zinc-mediated oxidative stress in metaphase II oocytes and embryos [83].

In bovine pre-implantation embryos, early embryonic development was analyzed with a wide range of R concentrations (36–7200 mg/L) that are usually used in agricultural practices, with an exposure time of 144 h (around 6 days). All doses were toxic to the embryos: the highest dose led to the death of the 2-cell embryo, 720 mg/L blocked the development of embryos at the 2- and 4-cell stages and then led to their death, and 36 mg/L stopped embryonic development at the compaction stage. Smaller doses were also assessed: At 7.2 mg/L of R, embryos survived the compaction stage, but for 7.2 and 3.6 mg/L of R, few embryos developed to the blastocyst stage. As the R concentration decreased (1.8, 0.9, 0.45 mg/L), the number of embryos reaching the blastocyst stage increased. After 84 h of exposure, increase Ca^2+^ and ROS amounts were observed with 0.45 mg/L of R as well as an increase in apoptosis. Interestingly, when the inner cellular mass (ICM) and trophectoderm (TE) were observed, there were no differences in the structure or the total number of cells [84].

Spinaci et al. [38] worked on an in vitro model of pig oocyte maturation. They analyzed the impact of exposure to G or R with the equivalent dose of G (0–360 mg/L). After the exposure, there were no effects on the percentage of oocytes fertilized and the cleavage rate after fertilization. However, 360 mg/L of G diminished the number of oocytes able to reach the blastocyst stage. At 200 mg/L, G negatively impacted the number of blastomeres per blastocyst. These effects were worse after exposure to R containing the equivalent dose of G. R exposure for 44 h induced a dose-dependent reduction in Pg (progesterone) as the R concentration increased. The differences between G and R exposures were multiple because G did not impact the intracellular ROS levels whereas R did. This study showed that G impaired early embryonic development (up to reaching the blastocyst stage), and R exposure exerted a more toxic effect than G likely because it contained formulants.

In an in ovo study in chickens, injection of 10 mg/kg of egg mass of G or R at growth day 6 did not impact the embryonic weight. However, there was a decrease in the hatching rate; an increase in kidney and liver weights and oxidative stress; serum parameter imbalances; histopathological alterations of the kidney, liver, and small intestine; and an increase in the levels of liver function enzymes. Moreover, some perturbations of cytochrome P450 family enzymes were recorded [85,86]. However, embryos exposed from the first day of incubation had a decreased bodyweight with another GBH: Fozat 480 at a higher dose (9.6 g/L). This treatment caused 40% embryo mortality [87].

Taken together, these studies have shown that GBHs could alter the early stages of embryonic development and R seems to be more toxic to embryonic cells than G alone.

### 7.2. In Vivo Studies

Exposure to G can directly affect in vivo embryonic development. Indeed, in a Japanese medaka model exposed to G or R for the first 15 days of their development, there were alterations in the mRNA levels of neuroendocrine hormones, with the expression of kiss and its receptors decreased after treatment with 0.5 or 5 mg/L of R. Expression of Dmrt1, a gene involved in sex differentiation and gonadal development, was also diminished in testes [20]. Webster et al. [88] showed that R exposure (10 mg/L) to zebrafish parents reduced the number of eggs spawned and increased early embryo mortality, early hatching, and developmental delay [88]. Moreover, changes in microtubule stability in zebrafish were observed after G exposure (5–50 mg/L) for 96 h post-fertilization. A reduction in the main component of microtubules, α-tubulin, in the polymer fraction was associated with an increase in the non-polymer fraction, an outcome that enhanced microtubule instability. Moreover, the acetylation status of α-tubulin was dysregulated. Histone deacetylase, the enzyme that deacetylates α-tubulin, is involved in processes like cell proliferation, metastases, invasion, etc., and can be mediated by the ER pathway. It also directly regulates the Wnt pathway, which is altered by GBHs, as we described above, and modulates α-tubulin acetylation [89]. The negative effect of G or R can be enhanced by the pH of the medium. An increased mortality rate has been observed when the pH was reduced to the same level as G. However, at pH 7, 84.5 mg/L of G led to developmental delay, heart rate acceleration, and induction of malformations in zebrafish [90].

An experiment using Xenopus showed that R was more toxic than other GBHs, as it reduced embryonic growth and increased teratogenic effects and malformations [91]. Paganelli et al. [91] worked on *Xenopus laevis* from the 2-cell stage to the neurula stage and showed a downregulation of neural crest marker (slug), the zinc finger transcription factor (krox-20), and N-tubulin, all important for the formation and differentiation of the neural system. They also measured the expression of morphogens like Shh (Sonic hedgehog), involved in developmental processes; Pax 6, essential for eye development; and otx2, important for the development of anterior structures. The expression of each was disturbed in a dose-dependent manner by GBHs (1/3500 or 1/4500 dilutions), causing teratogenic effects. They confirmed their results with a chicken model. Interestingly, they compared the G effects to retinoic acid pathway activation and found a similar effect, leading them to hypothesize that G uses this signaling pathway. When they used a retinoic acid receptor antagonist on chicken embryos exposed to G, they restored the control phenotype [91]. In another bird model, chronic exposure of Japanese quail females to R via their food (20 mg/kg/bw/d) triggered an egg-laying delay but did not impact the egg mass and the number of eggs [92].

In rodents, when mice were exposed to 5% of G or R from GD1 to GD19, at GD19, the fetus weight and the number of fetuses per dam were significantly decreased. Moreover, there was a reduction in the female/male ratio [22]. Teleken et al. [29] reported that exposure to R led to a delay in testis descent in male mice. In Sprague Dawley rats, pathways of NADPH generation in pregnant rats and their fetuses were altered after exposure to G (0.5% and 1%) [93]. The effects of the exposure to R (500 mg/kg) and another herbicide, paraquat (50 mg/kg), were also studied in 3-month-old female rats at different concentrations, administered together or separately. The results showed that the association of both herbicides affected embryonic development, with a disorganization of the cytotrophoblast and cell degeneration within the blastocyst cavity [92]. Malformations were also recorded in Wistar rat embryos when exposed to R during pregnancy, including incomplete skull ossification and unossified hind phalanges [94]. In humans, adverse pregnancy outcomes were reviewed but no significant link was reported between an increase in hypospadias, anencephaly, preterm delivery, birth weight, and exposure to G or R. However, exposure to G during the pre-conception period was related to late abortion. G has been detected in farmers’ urine in the µg/L range as well as in farmers’ children’s urine, but at lower concentrations [95].

GBHs have an impact from early embryonic development and exert negative effects in subsequent steps, such as neurulation and skull ossification. Although different effects occur according to the dose and the species considered, all information has confirmed that GBHs alter embryonic development.

## 8. Transgenerational Effects

The study of transgenerational effects comprises analysis of the indirect impacts of exposure to G or GBHs on subsequent generations (F1, F2, F3, etc.). This type of analysis often focuses on epigenetic modifications. Epigenetics is defined as “molecular factors and processes around DNA that regulate genome activity, independent of DNA sequence, and are mitotically stable.” Epigenetic modifications include DNA methylation, histone modification, non-coding mRNA, chromatin structure, and RNA methylation, among others. Several regions can be important in epigenetic regulation such as DNA methylation regions (DMRs) and differential histone retention regions (DHRs) [96].

In Japanese medaka, exposure to 0.5 mg/L of G or R altered epigenetic regulation in adults, with inhibition of *Dmnt1* transcription in the testes. This gene encodes an important factor that preserves methylation and regulates cell division. With this dysregulation, there was global hypomethylation [20]. In Japanese quails, parental exposure to a GBH (20 mg/kg/bw/d) for 12 months led to a tendency of poor embryonic development, increased lipid damage in the embryo’s brain, but no impact on the egg quality [92].

An epigenome-wide association study was performed to analyze the transgenerational impact of G exposure on sperm DNA. This experiment consisted of the exposure of female F0 mice during pregnancy (25 mg/kg/bw/d) from GD8 then analyzing the three next generations. F1 was directly exposed to G in utero and F2 was exposed through the F1 germline; however, F3 was not exposed directly, so they would potentially manifest transgenerational effects. In male F3 mice, there was increased obesity with 250 differential DMRs (and the presence of testis and kidney diseases (with 180 differentially methylated DMRs) and prostate diseases (with 242 differentially methylated DMRs). In the testis, there was atrophy of seminiferous tubules with vacuoles and arrested maturation. In the prostate, there was atrophy of the epithelium with vacuoles and hyperplasia [96]. Among the differentially methylated DMRs, about 5% had an increase in DNA methylation and 50% had a decrease in DNA methylation. Moreover, the study showed that several DHRs were located in sperm and provided biomarkers for diseases. For females, there was an increased pathology rate in ovaries from F2 and F3 mice (polycystic ovary syndrome, altered granulosa cells, etc.), more parturition issues, and general pathology (hepatic necrosis, adrenal cortical necrosis, etc.) [97]. These two studies demonstrated that G acts through epigenetic dysregulation by inheritance to exert its negative effects, and these effects are more important in the offspring than in the initial generations exposed to the treatment.

Perinatal GBH exposure (350 mg/kg/bw/d) led to implantation failures by changing the Erα epigenetic regulation within the uterus of F1 Wistar rats. Erα transcription is controlled by five promoters (EI, OT, O, ON, and OS) corresponding to different transcript variants. The GBH induced the transcription of Erα mRNA by increasing the Era-O transcript variant. Indeed, the GBH decreased DNA methylation, increased acetylation of histone H4 and histone H3 lysine 9 trimethylation (H3K9me3), and finally reduced H3 lysine 27 trimethylation (H3K27me3) in the O promoter [98]. Moreover, there were adverse effects during pregnancy of F1 generation, denoted by fewer implantation sites, increased pre-implantation loss, and altered F2 offspring fetal development associated with a decrease in height and weight and an increase in placental height and fetal malformations [99]. In rats, male mammary gland development was perturbed by GBH exposure (Magnum super II) during pregnancy (0, 3.5, and 350 mg/kg/bw/d) from GD9 to weaning. At PND60, there was decreased *Esr1* mRNA and protein in relation to hypermethylation of the promoter in the ESR1-OS region for 3.5 mg/kg/bw/d and ESR1-O, OT, and EI at 350 mg/kg/bw/d [100]. Maamar et al. [96] performed an epigenome-wide association study between exposure to G and male fertility and found histone retention epigenetic biomarkers for obesity, testicular diseases, and prostate diseases in the F3 mouse generation.

G can be absorbed through feeding, including soybeans. Brake et al. [101] used glyphosate-tolerant soybeans (the ration contained 21.35% glyphosate-tolerant soybeans by weight) to feed mice during gestation and weaning and the F1 offspring for 87 days. The authors observed the fourth generation (F3). There were no relevant findings regarding the percentage of cells in the testicular population, body weight, spermatogenesis, and spermiogenesis. Therefore, glyphosate-tolerant soybeans did not have negative effects on fetal, postnatal, pubertal, and adult testicular development [101].

An in vitro study performed using human PBMCs showed that G and AMPA exposure (0.5–100 µM and 0.5–250 µM, respectively) altered DNA methylation by increasing DMNT1 and DNMT3A expression. In addition, exposure to 0.5 µM of G or 250 µM of AMPA for 24 h perturbed histone deacetylation, a phenomenon associated with an increase in histone deacetylase 3 (HDAC3). There were no effects reported about DNA demethylation and chromatin remodeling [102].

Taken together, G and GBHs have transgenerational effects that perturb fetal development but also adult reproductive life by modifying methylation patterns of important genes involved in reproduction such as Erα.

## 9. Involvement of Glyphosate or GBH in Reproductive Pathologies

As described before, G and GBHs exert adverse effects on fertility and induce alterations at different levels of the HPG axis. However, it is still unclear how GBHs are associated with reproductive pathologies. First, traces of G have been found in human urine in the µg/L range [8], with the highest concentration of 7.6 µg/L. In serum from women and umbilical cord serum, G has also been measured in the µg/L range [80]. This proves that GBHs are absorbed and metabolized by humans, so they could have an impact on human health.

Researchers have evaluated the adverse pregnancy outcomes due to exposure to G. De Araujo et al. [95] reported a non-significant association between G or GBH parental exposure and an increase in hypospadias, anencephaly, gastroschisis, preterm deliveries, congenital anomalies, and birth weight. There was a possible link between G exposure during preconception and the risk of late abortion [91]. Multiple observational studies have been performed on people living near agricultural areas. For example, Garry et al. [103] examined a population of 695 families and 1532 children living in the Red River Valley in Minnesota, United States, between 1997 and 1998. This population attracted attention because of its high rate of birth defects (31.3 per 1000 live births) during the first year of the study and the high rate of children with developmental disorders in the first 3 years of life (47 per 1000 live births). Moreover, conceptions occurring during spring involved more children with birth defects than in other seasons. This study found a possible link between G exposure and attention deficit disorder with or without attention deficit hyperactivity disorder [103]. The atrial septal defect was positively associated with exposure to a high dose of G in a 2003–2005 North Carolina birth cohort [104]. Exposures to GBHs were also analyzed in an Ontario farm family health study during the peri-conception period. There was a non-significant association found between GBHs and miscarriages, preterm deliveries, small-for-gestational-age births, and sex ratios [105]. However, a study highlighted a possible link between preconception exposure to glyphosate and spontaneous abortion [106].

G and GBHs increase oxidative stress imbalance, and this change could lead to pathogenesis of subfertility issues. Some known reproductive pathologies such as endometriosis are associated with high oxidative stress. This pathology is related closely to the quality of life for women and their reproduction capacities. This estrogen-dependent, inflammatory syndrome causes chronic pelvic pain and affects around 10% of women of childbearing age [107]. Paris et al. [107] reviewed the possible link between genetically modified food (GMF) with xenobiotic association and endometriosis. They argued two hypotheses for the origins of endometriosis (i) a retrograde menstruation of endometrial cells in the peritoneal cavity and (ii) an alteration in the immune system leading to excessive receptivity of endometrium, hyperactivity of macrophages, and natural killer (NK) cell anomalies. Moreover, they proposed “the low dose hypothesis”: At a low dose, some environmental chemicals have greater effects on human health if people are exposed to it daily, and the chemicals perturb their endocrine system, whereas acute exposure to high doses has no effect. To support this, they reported that some pesticides decrease NK cell activity, phagocytosis of neutrophils, and the proliferative response of lymphocytes, leading to disturbed immunomonitoring. Finally, Paris et al. [107] recorded studies that worked on the association between pesticides, increased ROS levels, and fertility impairment. As reported before, G and GBHs disturb steroidogenesis, uterine development and regulation, and embryonic development. Important hormones for uterine development are estrogen and Pg. Aromatase converts androgen into estrogen. They reported that an important activity of aromatase could be involved in the pathology of endometriosis because a large amount of estrogen is observed in this syndrome and pesticides such as R target aromatase. To strengthen this last idea, *Ers2* and *Cyp19a1* genes have been reported as implicated in endometriosis [107]. Local increases in estrogen production in the endometrium, related to an increase in aromatase, were reported with epigenetic regulation in this review. Moreover, the main ER involved in endometriosis is ERβ. This is in accordance with Guerrero-Schimpf et al. [74], who recorded an upregulation of esr2 associated with cell proliferation and hyperplastic uterus after G exposure. A high level of ROS has also been reported in endometriosis [108]. Oxidative balance is crucial for the proper function of the estrus cycle. The selection of the dominant oocyte and meiosis I are regulated by a high level of ROS, which inhibits antioxidants. On the contrary, meiosis II needs a low ROS level and a high amount of antioxidants. In endometriosis, high concentrations of MDA and oxidized LDL (Low-Density Lipoprotein) were detected within the peritoneal fluid. We can hypothesize that G exposure exacerbates oxidative imbalance leading to the inflammatory status of the endometrium and worsening endometriosis.

Exposure to G could also be related to polycystic ovary syndrome (PCOS), which includes hyperandrogenism, ovulatory dysfunction, and polycystic ovaries. It is associated with a decrease in antioxidant concentrations, mitochondrial O2 consumption, and an increase in ROS. These deleterious effects are equivalent to those found under G exposure in human cells [71,79,108].

Many cases of idiopathic infertilities are related to increased ROS levels. Chronic exposure to pesticides through agricultural activities or food containing R could be related to these unexplained fertility issues [108]. Although only a few studies have been performed to evaluate how exposure to G or GBHs could be related to infertility, there is evidence that GBH exposure impacts human health. GBHs have been related to breast cancer. Indeed, the development of the mammary gland depends on estrogen and Pg secretion. GBHs disturbed mammary gland development and the expression of steroid hormone receptors, with an increase in cell proliferation in male rats when they were exposed perinatally and postnatally [109]. Moreover, in a recent study, maternal exposure to G has been related to an increase in the case of autistic behavior [110] and celiac diseases [111]. The latter study also reported impairment in many cytochrome P450 enzymes, some infertility issues, miscarriages, and birth defects.

## 10. Conclusions and Perspectives

Here, we have reported that G acts at the hypothalamic, pituitary, gonadal, uterine, placental, and embryonic development levels. Moreover, alterations can be transmitted to the next generations through epigenetics effects and so the fertility of offspring is not protected against the negative effects of GBHs. All studies presented here show the higher impact of GBHs containing formulants compared to G alone. G acts as an EDC because it alters GnRH, LH, FSH, E2, and Pg secretions and the activity of steroidogenic enzymes. Moreover, it seems to act differently depending on the sex of the organism. In males, StAR, a cholesterol carrier, is more affected than other actors of steroidogenesis, whereas, in females, aromatase is also strongly affected. Finally, G has a negative impact on oxidative balance and increases ROS levels and lipid peroxidation, and decreases mitochondrial respiration. These perturbations might lead to reproductive pathologies such as endometriosis or PCOS, or they might be the origin of idiopathic reproductive pathologies. However, such effects could be prevented by protective molecules. Indeed, some studies have attempted to find ways to decrease the impact of herbicides. Co-administration of alpha-lipoic acid with GBHs in Wistar rat restored T, LH, FSH, 3βHSD, and 17βHSD levels [24]. Resveratrol, an antioxidant, was co-administered with G and increased sperm motility, decreased the abnormal sperm rate, and restored ROS levels [65]. Melatonin has been proven to be a good protective molecule against G when co-administrated to females, with inhibition of apoptosis and autophagy, normalization of ROS levels, increased ovary weight, and the number of corpus lutea, and better viability of implanted embryos [92,112]. Another antioxidant, Dig1, has appeared as a good candidate to prevent G-mediated alterations when it was co-administrated with GBHs because it restored plasma E2 levels in vivo [64] and avoided alterations in cytochrome P450s, toxic effects, and apoptosis in in vitro human liver cell line study [113]. Vitamins C and E (antioxidants), quercetin (mitochondria-targeted flavonoid), and fulvestrant (ER antagonist) have also been tested in studies focusing on antral follicles of goats, prepubertal testes, human sperm, and human endometrial cells, respectively. These molecules showed potent protective effects against exposure to G or GBHs, with decreased apoptosis, normalization of ROS levels and mitochondrial respiration, and reduced lipid peroxidation and Ca^2+^ uptake [42,66,71,78,113].

Finally, GBHs were found to be more deleterious than G alone, undoubtedly related to the presence of formulants such as POEA [63]. A higher dose of G alone is needed to reach the same effect of the R formulation. More studies should be performed to determine the impact of GBHs on fertility and the associated molecular mechanisms.

In conclusion, G or GBHs can induce alterations in all the reproductive tracts in both males and females. These alterations could be prevented or reduced with protective molecules (Table 7). However, more studies need to be performed to analyze the impacts of GBH exposure on human fertility, the consequences on human health in terms of reproductive pathologies, the transgenerational effects, and, finally, to find strategies to decrease or to avoid the negative impacts of GBHs.

## Figures and Tables

**Table 1:** Summary of studies on the impact of G or GBH exposure on the pituitary F: female; M: male; Eq: equivalent. Numbering in brackets indicates references.

Hormone	Pesticide	Doses Eq. G	Sex	Species	Stages	Time Exposure	Compartment	Molecules	Effect	References
FSH	R	10–40 mg/kg/bw/d	F	Pig	weaned	36 days	Serum	Hormone	↘	[21]
	R	0.5%	F	ICR mice (Pregnant)	10 weeks	GD1-GD19	Pituitary	mRNA	↗	[22]
	R	50 mg/kg/bw/d	M	Wistar rat	60 days	GD18-PND5	Pituitary	mRNA	↗	[28]
	G	5 mg/kg *	M		6 weeks	52 days	Plasma & Pituitary	Hormone	NS	[25]
	G	10 mg/kg/bw	M		2 months (adult)	3 times a week during 5 weeks	Plasma	Hormone	↗	[24]
	R	400 or 2000 mg/kg/bw	M		Adult	60 days	Pituitary	Hormone	↘	[23]
	R	1.85 g/L **	M	C57Bl/6 mice	50 days	GD4-PND30	Plasma & Pituitary	Hormone	NS	[29]
	R	3.6–248.4 mg/kg/bw	M	Albino rat	Adult	12 weeks	Blood	Hormone	↘	[26]
LH	R	0.5%	F	ICR mice (Pregnant)	10 weeks	GD1-GD19	Pituitary	mRNA	↗	[22]
	R	50 mg/kg/bw/d	M	Wistar rat	60 days	GD18-PND5	Pituitary	mRNA	↗	[28]
								Hormone	↗	
	G	5 mg/kg	M		6 weeks	52 days	Plasma & Pituitary	Hormone	NS	[25]
	G	10 mg/kg + other pesticides	M		2 months	3 times a week during 5 weeks	Plasma	Hormone	↗	[24]
	R	400 or 2000 mg/kg	M		Adult	60 days	Pituitary	Hormone	↗	[23]
	R	3.6–248.4 mg/kg	M	Albino rat	Adult	12 weeks	Blood	Hormone	↘	[26]

* kg of corn oil; ** distilled water.

**Table 2:** Summary of in vivo studies on the impact of G or GBH exposure on steroidogenesis in females. Numbering in brackets indicates references.

Pesticides	Doses Eq. G	Time Exposure	Species	Stages	Compartment	Hormones	References
R	10 mg/kg/bw	36 days	Pig	weaned	plasma	Estrogen ↘; LHRH ↗; Testosterone ↗; Prolactin ↘	[21]
R	65 µg/L *	15 days	Zebrafish		ovary	SF-1 (steroidogenic factor-1) ↗	[ 39]
R	2 mg/kg/day	15 days	Ovine	PND1—PND14	granulosa	*FSHR* *↘; GDF9* *↘*	[40]
R	1.75 mg/kg/bw/day	GD6—PND120	Sprague Dawley rats	pregnancy and weaning		Estrogen NS	[31]
R	5 g/L **	GD1—GD19	ICR mice (Pregnant)	pregnancy	serum	Pg ↘	[22]
					ovary	*LHR* ↘; 3β HSD ↘; Cyp17a1 ↗	
G	5 g/L **		ICR mice (Pregnant)		serum	Estrogen ↗; Pg ↘; FSHR ↘; Cyp11a1 ↗; 3β HSD ↘; Cyp19a1 ↗	
K360	126 mg/kg **	60 days	Wistar rat		ovary	Estrogen ↘	[33]
G	2 mg/kg/bw 5 times a week	20 weeks	C57BL6 mice	6 weeks old		Estrogen NS; NS Progesterone	[32]

K360: Kalach 360 SL, Eq: equivalent; * aquarium water; ** drinking water.

**Table 3:** Summary of in vitro studies on the impact of G or GBH exposure on steroidogenesis in females. Eq: equivalent. Numbering in brackets indicates references.

Pesticides	Doses Eq. G	Time Exposure	Species	Stages	Compartment	Hormones	References
R + FSH + T + IGF1	10 mg/L or 300 mg/L	2 days	Bovine		granulosa	Estrogen ↘; Pg ↘	[35]
R + FSH + T+ IGF1	10 mg/L					Estrogen ↘; Pg ↘	
R + FSH + T + IGF1	1 mg/L					Estrogen ↗; Pg ↗	
R + FSH + T	1 mg/L					Estrogen ↘	
R	10 µg/L	72 h	Bovine	8th & 12th days of estrus cycle	granulosa	Estrogen NS	[37]
G	10 µg/L		Bovine			Estrogen ↗	
G	200 µg/L	48 h	Pig		granulosa	Estrogen ↘; Pg ↗	[30]
G + FSH + IGF1	5 mg/L	2 days	Bovine		granulosa	Estrogen ↘; Pg NS	[36]
	10 mg/L or 300 mg/L					NS	
G	200 mg/L	44 h	Pig		oocyte	Pg ↗	[38]
R	100 mg/L					Pg ↘	

**Table 4:** Summary of in vivo studies of G or GBH exposure impact on testosterone secretion. * drinking water; Eq: equivalent. Numbering in brackets indicates references.

Pesticide	Doses Eq. G	Time Exposure	Species	Stages	Compartment	Effect	References
R	50- 450 mg/kg/bw/d	GD21-PND21	Wistar rat	puberty		↘	[45]
R	50 mg/kg/bw/d	GD18-PND5	Wistar rat	adult		↗	[28]
R	1.85 g/L *	GD4-PND30	C57 Bl/6 mice			↗	[29]
R	5–250 mg/kg/bw/d	30 days	Wistar rat	prepubertal		↘	[46]
					serum		[47]
R	3.6–248.4 mg/kg/bw/d	12 weeks	Albino rat	adult	blood	↘	[26]
		15 days	Drakes	adult	serum		[53]
		6 weeks	Rabbit	adult			[56]
G	5–125 mg/kg/bw/d	10–20 days	Wistar rat	adult	serum	↘	[48]
		52 days	Wistar rat	adult	plasma	↘	[25]
		5 weeks	Wistar rat	adult	plasma	↘	[24]
		60 days	Wistar rat	adult		↘	[23]
		4 weeks	BALB/c mice	adult	serum	↘	[57]
G	0.5–50 mg/kg/bw/d	GD10.5-PN20	Swiss mice	PND5-8 months		↘	[54]

**Table 5:** Summary of in vitro studies of G or GBH exposure impact on testosterone secretion. Eq: equivalent. Numbering in brackets indicates references.

Pesticide	Doses Eq. G	Time Exposure	Species	Stages	Compartment	Effect	References
R	360 µg/L	24 h	Albino Sprague Dawley rats	adult	Rat testis	↘	[58]
G	360 µg/L					↘	
	5 mg/L	1–24 h	Mouse		TM3 mouse Leydig cells	↘	[59]
	16.9 mg/L	24 h	Mouse		TM3 mouse Leydig cells	↘	[57]
	16.9 mg/L		Mouse		Primary mouse Leydig cells	↘	

**Table 6:** Summary of studies on the impact of G or GBH exposure on steroidogenesis in adult males. Numbering in brackets indicates references.

Experiment	Pesticide	Doses Eq. G	Time Exposure	Species	Compartment	Hormones	Effect	References
In vivo	G	5 g/L *	4 weeks	BALB/c mice	serum	StAR	↘	[57]
						*StAR* mRNA	↘	
	G	2.5 mg/L *	2 weeks	Sprague Dawley rats	testis	StAR	NS	[51]
						*StAR* mRNA	↘	
In vitro	G	5 mg/L	1–24 h	Mouse	TM3 mouse Leydig cells	StAR	↘	[59]
	G	16.9 mg/L	24 h		TM3 mouse Leydig cells	StAR	↘	[57]
						*StAR* mRNA	↘	
	R	24 mg/L	4 h		Mouse MA-10 Leydig Tumor cell line	StAR	↘	[60]
						*StAR* mRNA	NS	
In vivo	R	2.25 g/L *	8 days	Sprague Dawley rats		*CYP19A1*	↗	[49]
In vitro	G	360 µg/L	24 h	Albino Sprague Dawley rats	testis	*CYP19A1*	↘	[58]
	R	360 µg/L					↘	
	R	2.16 g/L	18 h	Equine	testis	P450 aromatase	↘	[61]
	G	6.48 g/L					↘	[62]

* Drinking water. Eq: equivalent; TM3 and MA-10: strain of mouse cell line.

**Table 7:** Summary of studies on protective molecules against GBH exposure impact on fertility. M: male; F: female; PM: protective molecules; ROS: Reactive Oxygen Species; Cat: Catalase, MDA: Malondialdehyde; SOD: Superoxide dismutase; GST: glutathion S-transferase, GPx: Glutathion Peroxidase. Numbering in brackets indicate references.

Experiment	Pesticides	Doses	Protective Molecules (PM)	Dose (PM)	Time Exposure	Sex	Species	Stages	Steroidogenesis	Oxidative Stress	Sperm	Other Effects	References
In vivo	Mixture (Zineb+G+Dimethoat)	10 mg/kg/bw	α-Lipoic acid	50 mg/kg/bw/d	5 weeks	M	Wistar rat	8 weeks	Restored T, LH, FSH, 3 and 17 βHSD level				[24]
	G	375 mg/kg/bw/d	Resveratrol	20 mg/kg/bw/d	8 weeks	M	Wistar Albino rat	12 weeks		Restored MDA level	↗ sperm motility ↘ abnormal sperm rate no effect on sperm concentration	↗ plasma integrity, ↘ DNA damage, no effect on Sertoli cells	[65]
	G	250–500 mg/kg/bw/d	Melatonin	15 mg/kg	7 days	F	Kumming mice	4 weeks		Normalized Cat, GPx activity, restored mitochondrial ATP, ↘ ROS levels		Enhance meiotic progression, normalize Bax/Bcl2, inhibition of apoptosis and autophagy	[112]
	R	500 mg/kg/bw	Melatonin	10 mg/kg	GD1-GD7	F	Wistar rat	12 weeks				No effect on the morphology of blastocysts, ↗ weight of ovary and number of corpus lutea, ↗ viability of the embryo implantation	[92]
	R	135 mg/kg/bw/d	Dig1	1.2 mg/kg/bw/d	8 days	M	Sprague Dawley rat	adult	Restored plasma estradiol level				[64]
	R	50 mg/kg/bw/d	Ginkgo Biloda (leaf extract)	50 o 150 mg/kg/bw/d	72 h	/	Swiss Albino mice	12–14 weeks		Restored ROS level in kidney and liver		↘ apoptosis	[113]
In vitro	R	0.1–4.0 g/L	Vitamin C	1 mM	24–72 h	F	Caprine	Antral follicle		Restored MDA and ROS levels, normalized Cat, SOD and GST activity			[42]
	R	0.72–360 mg/L	Vitamin C + Trolox (Vit E) Nifedipine	100 μM + 100 μM 10μM	30 min	M	Wistar rat	Prepubertal testis				↘ Ca^2+^ uptake	[66]
	G	0.069–169 mg/L	Quercetin	1 nM	10 min	M	Human sperm			Normalized mitochondrial respiration			[71]
	G	33.8–338.1 μg/L	Fulvestrant (ER antagonist)	700 nM	24 h	F	Human endometrial Ishikawa cells					Normalized endometrial cell migration and invasion, normalized level of E-Cadherin	[78]

## Data Availability

Not applicable.

## Abbreviations

3βHSD	3 β-hydroxysteroid dehydrogenase
ADI	Acceptable daily intake
AOEL	Acceptable operator exposure level
AM	Antimesometrial
AMPA	Amino-methyl-phosphonic acid
ATP	Adenosine triphosphate
BTB	Blood-testis barrier
Ca^2+^	Calcium
cAMP	Cyclic adenosine 3′,5′-monophosphate
CAT	Catalase
COCs	Cumulus-oocyte complexes
CYP11a1	Cytochrome P450 family 11 subfamily A member 1
CYP17a1	Cytochrome P450 family 17 subfamily A member 1
CYP19a1	Cytochrome P450 family 19 subfamily A member 1
Δ4	Androstenedione
DDI	Dietary Daily Intake value
DHEA	Dehydroepiandrosterone
DHR	Differential histone retention region
Dmnt1	DNA (cytosine-5)-methyltransferase 1
Dmrt1	Doublesex and mab-3 related transcription factor 1
DMR	DNA methylation region
E2	Estradiol
EC	Endometrial cells
ECM	extracellular matrix
ED	Endocrine disruptors
EDC	Endocrine-disrupting chemical
EFSA	European Food Safety Authority
EPSPS	5-enolpyruvylshikimate-3-phosphate synthase
ER	Estrogen receptor
FAO	Food and Agricultural Organization of the United Nation
Foxa2	Forkhead box a2
FSH	Follicle-stimulating hormone
G	Glyphosate
GBH	Glyphosate-based herbicide
GC	Granulosa cells
GD	Gestational day
GDF9	Growth differentiation factor 9
GE	Glandular epithelium
GnRH	Gonadotropin-releasing hormone
HDAC3	Histone Deacetylase 3
HHR	Hypothalamic-Pituitary-Renal
Hox a10	Homeobox a10
HPG	Hypothalamic-pituitary-gonadal
HPT	Hypothalamic-Pituitary-Thyroid
ICM	Inner cellular mass
IGF1	Insulin-like growth factor 1
Insl3	Insuline-like-3
IP3	Inositol triphosphate
KCs	Key characteristics
Kiss1	Kisspeptin protein
LA	Alpha-lipoic acid
LC	Leydig cells
LE	Uterine luminal epithelium
LH	Luteinizing hormone
M	Mesometrial
MDA	Malondialdehyde
mg/kg/bw/d	mg/kg bodyweight/day
MMP	Mitochondrial membrane potential
MRL	Maximum Residues Limits
MTOC	MicroTubule-Organizing Center
NADPH	Nicotinamide adenine dinucleotide phosphate
NO	Nitrogen oxide
NOAEL	No observed adverse effect level
NR1D1	Nuclear receptor subfamily 1, group D member 1
PBMC	Peripheral blood mononuclear cell
PCOS	Polycystic ovary syndrome
P450 scc	Sidechain cytochrome P450
Pg	Progesterone
PI3K	Phosphoinositide 3-kinase
PKA	Protein Kinase A
PKC	Protein Kinase C
PM	Pesticide mixture
PND	Post-natal day
POA	Preoptic area
POEA	Polyoxyethylene tallow amine
SS	Endometrial stroma
R	Roundup
ROS	Reactive oxygen species
SC	Sertoli cells
SOD	Superoxide dismutase
ST	Seminiferous tube
StAR	Steroidogenic Acute Regulatory protein
T	Testosterone
TE	Trophectoderm
TH	Thyroid hormone
